# 2,4,6-Triphenyl-*N*-{(3*E*)-3-[(2,4,6-tri­phenyl­phen­yl)imino]­butan-2-yl­idene}aniline

**DOI:** 10.1107/S2414314620005313

**Published:** 2020-04-30

**Authors:** Yang Zhang, Fan Yu, Pei Li, Mengli Xu, Guoyong Xu, Weimin Li, Fuzhou Wang

**Affiliations:** a School of Petrochemical Engineering, Changzhou University, Changzhou 213164, People’s Republic of China; b Institutes of Physical Science and Information Technology, Anhui University, Hefei 230601, Anhui, People’s Republic of China; Sunway University, Malaysia

**Keywords:** crystal structure, *C_i_* symmetry, 1,4-di­aza­butadiene

## Abstract

The title compound, C_52_H_40_N_2_, is disposed about a centre of inversion and the conformation about the imine bond [1.268 (3) Å] is *E*. The terminal benzene ring is approximately perpendicular to the central 1,4-di­aza­butadiene mean plane, forming a dihedral angle of 81.2 (3)°. Weak C—H⋯π and π–π [inter-centroid distance = 4.021 (5) Å] inter­actions help to consolidate the packing.

## Abstract 2

The title mol­ecule is disposed about a centre of inversion and the conformation about the imine bond is *E*. The terminal benzene ring is approximately perpendicular to the central 1,4-di­aza­butadiene mean plane, forming a dihedral angle of 81.2 (3)°.

## Graphical abstract

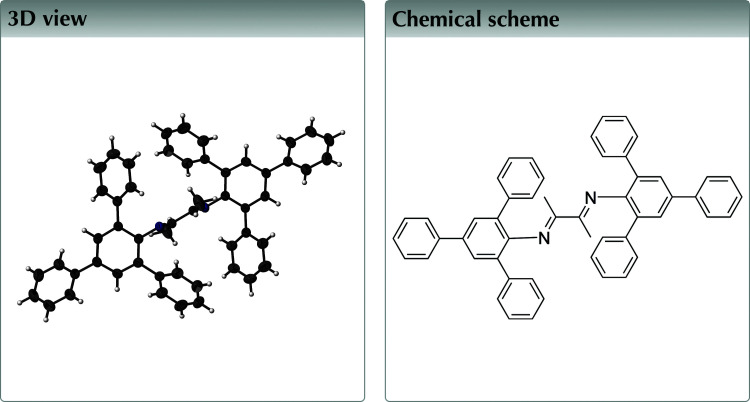

## Structure description

The seminal studies by Brookhart and co-workers leading to the discovery of cationic α-di­imine-based Ni and Pd catalysts marked the start of a new era in olefin polymerization studies (Killian *et al.*, 1996 ▸). Branched polyolefins are generally produced using these catalysed ethyl­ene polymerizations through a characteristic chain-walking process (Wang & Chen, 2019 ▸). More importantly, these α-di­imine Ni and Pd catalysts are able to co-polymerize olefins with polar co-monomers to afford co-polymers containing functional groups without the pre-protection of the polar groups (Chen *et al.*, 2018 ▸). For the synthesis of the α-di­imine mol­ecules and background to the applications of the olefin polymerization catalysts ligated by α-di­imine, see: Wang *et al.* (2016 ▸, 2018 ▸, 2019 ▸).

**Figure 1:**
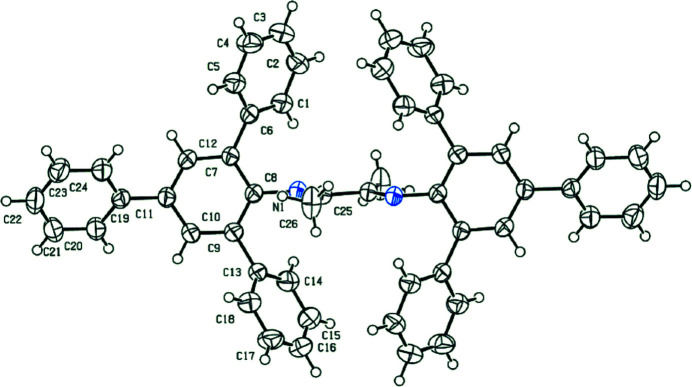
Mol­ecular structure of the title compound showing the atom-labelling scheme and displacement ellipsoids at the 30% probability level. Unlabelled atoms are related by the symmetry operation − *x*, 2 − *y*, 1 − *z*.

In this study, we designed and synthesized the title compound (Fig. 1 ▸) as a potential bidentate ligand, and its mol­ecular structure was characterized by X-ray diffraction. In the solid state, the molecule exhibits *C_i_* symmetry, being disposed about a centre of inversion. The single bond of the 1,4-di­aza­butadiene fragment [1.491 (4) Å] has an anti-disposition and the imine bonds [1.268 (3) Å] are *E*-configured. The dihedral angle between the pendent benzene ring and the 1,4-di­aza­butadiene least-squares plane is 81.2 (3)°, consistent with an almost perpendicular relationship. In the crystal, C—H⋯π, Table 1 ▸, and π–π inter­actions are noted. For the latter, the closest approach of 4.021 (5) Å occurs between centrosymmetrically related (C13–C18)-phenyl rings with the off-set distance being 1.86 Å; symmetry operation −*x*, 1 − *y*, 1 − *z*.

## Synthesis and crystallization

**Figure 2:**
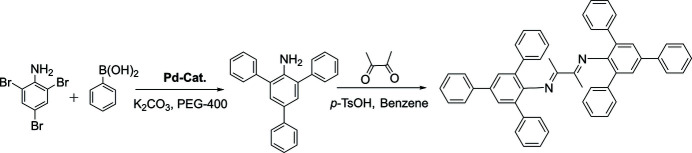
Reaction scheme.

After the protection of the amino group by acetic acid, the aniline was brominated. The Suzuki coupling reaction of the aniline and phenyl­boronic acid catalysed by a Pd catalyst in PEG-400 /H_2_O led to the corresponding triphenyl-substituted aniline (Fig. 2 ▸). The title compound was prepared by the condensation of two equivalents of the appropriate aniline with one equivalent of 2,3-butane­dione, in the presence of formic acid or *p*-toluene­sulfonic acid, as a catalyst at 81% yield.

## Refinement

Crystal data, data collection and structure refinement details are summarized in Table 2 ▸.

## Supplementary Material

Crystal structure: contains datablock(s) I, New_Global_Publ_Block. DOI: 10.1107/S2414314620005313/tk4062sup1.cif

L1-cif. DOI: 10.1107/S2414314620005313/tk4062sup3.txt

CCDC reference: 1949863

Additional supporting information:  crystallographic information; 3D view; checkCIF report

## Figures and Tables

**Table 1:** Hydrogen-bond geometry (Å, °) *Cg*1 is the centroid of the (C19–C24) phenyl ring.

*D*—H⋯*A*	*D*—H	H⋯*A*	*D*⋯*A*	*D*—H⋯*A*
C17—H17⋯*Cg*1^i^	0.93	2.89	3.737 (5)	152

Symmetry code: (i) 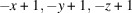 .

**Table 2:** Experimental details

Crystal data	
Chemical formula	C_52_H_40_N_2_
*M*_r_	692.86
Crystal system, space group	Triclinic, *P* 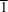
Temperature (K)	296
*a*, *b*, *c* (Å)	6.383 (8), 12.498 (15), 12.814 (16)
α, β, γ (°)	68.718 (11), 86.988 (12), 81.397 (12)
*V* (Å^3^)	942 (2)
*Z*	1
Radiation type	Mo *K*α
μ (mm^−1^)	0.07
Crystal size (mm)	0.23 × 0.21 × 0.20

Data collection	
Diffractometer	Bruker APEXII CCD
Absorption correction	Multi-scan (*SADABS*; Sheldrick, 1996 ▸)
*T*_min_, *T*_max_	0.984, 0.986
No. of measured, independent and observed [*I* > 2σ(*I*)] reflections	6847, 3456, 2023
*R*_int_	0.049
(sin θ/λ)_max_ (Å^−1^)	0.606

Refinement	
*R*[*F* ^2^ > 2σ(*F* ^2^)], *wR*(*F* ^2^), *S*	0.060, 0.158, 1.02
No. of reflections	3456
No. of parameters	245
H-atom treatment	H-atom parameters constrained
Δρ_max_, Δρ_min_ (e Å^−3^)	0.16, −0.20

Computer programs: *APEX2* and *SAINT* (Bruker, 2002 ▸), *SHELXS97* (Sheldrick, 2015*a* ▸), *SHELXL97* (Sheldrick, 2015*b* ▸) and *SHELXTL* (Sheldrick, 2008 ▸).

## full crystallographic data

### Crystal data

**Table:**

C_52_H_40_N_2_	*Z* = 1
*M**_r_* = 692.86	*F*(000) = 366
Triclinic, *P*1	*D*_x_ = 1.222 Mg m^−^^3^
*a* = 6.383 (8) Å	Mo *K*α radiation, λ = 0.71073 Å
*b* = 12.498 (15) Å	Cell parameters from 936 reflections
*c* = 12.814 (16) Å	θ = 2.9–21.8°
α = 68.718 (11)°	µ = 0.07 mm^−^^1^
β = 86.988 (12)°	*T* = 296 K
γ = 81.397 (12)°	Block, yellow
*V* = 942 (2) Å^3^	0.23 × 0.21 × 0.20 mm

### Data collection

**Table:**

Bruker APEXII CCD diffractometer	3456 independent reflections
Radiation source: fine-focus sealed tube	2023 reflections with *I* > 2σ(*I*)
Graphite monochromator	*R*_int_ = 0.049
φ and ω scans	θ_max_ = 25.5°, θ_min_ = 2.9°
Absorption correction: multi-scan (SADABS; Sheldrick, 1996)	*h* = −7→7
*T*_min_ = 0.984, *T*_max_ = 0.986	*k* = −15→15
6847 measured reflections	*l* = −15→15

### Refinement

**Table:**

Refinement on *F*^2^	Primary atom site location: structure-invariant direct methods
Least-squares matrix: full	Secondary atom site location: difference Fourier map
*R*[*F*^2^ > 2σ(*F*^2^)] = 0.060	Hydrogen site location: inferred from neighbouring sites
*wR*(*F*^2^) = 0.158	H-atom parameters constrained
*S* = 1.02	*w* = 1/[σ^2^(*F*_o_^2^) + (0.0398*P*)^2^] where *P* = (*F*_o_^2^ + 2*F*_c_^2^)/3
3456 reflections	(Δ/σ)_max_ < 0.001
245 parameters	Δρ_max_ = 0.16 e Å^−^^3^
0 restraints	Δρ_min_ = −0.19 e Å^−^^3^

### Special details

**Table:**

Geometry. All esds (except the esd in the dihedral angle between two l.s. planes) are estimated using the full covariance matrix. The cell esds are taken into account individually in the estimation of esds in distances, angles and torsion angles; correlations between esds in cell parameters are only used when they are defined by crystal symmetry. An approximate (isotropic) treatment of cell esds is used for estimating esds involving l.s. planes.
Refinement. Refinement of F^2^ against ALL reflections. The weighted R-factor wR and goodness of fit S are based on F^2^, conventional R-factors R are based on F, with F set to zero for negative F^2^. The threshold expression of F^2^ > 2sigma(F^2^) is used only for calculating R-factors(gt) etc. and is not relevant to the choice of reflections for refinement. R-factors based on F^2^ are statistically about twice as large as those based on F, and R- factors based on ALL data will be even larger. All hydrogen atoms were placed in calculated positions with C—H distances of 0.93 and 0.96 Å for aryl and methyl type H-atoms. They were included in the refinement in a riding model approximation, respectively. The H-atoms were assigned *U*iso = 1.2 times *U*eq of the aryl C atoms and 1.5 times *U*eq of the methyl C atoms.

### Fractional atomic coordinates and isotropic or equivalent isotropic displacement parameters (Å^2^)

**Table:**

	*x*	*y*	*z*	*U*_iso_*/*U*_eq_	
C1	0.1450 (4)	0.9506 (2)	0.8008 (2)	0.0489 (7)	
H1	0.0143	0.9357	0.7848	0.059*	
C2	0.1537 (5)	1.0422 (2)	0.8349 (2)	0.0596 (8)	
H2	0.0291	1.0883	0.8417	0.071*	
C3	0.3434 (5)	1.0660 (2)	0.8588 (2)	0.0632 (8)	
H3	0.3483	1.1280	0.8821	0.076*	
C4	0.5269 (5)	0.9980 (2)	0.8481 (2)	0.0616 (8)	
H4	0.6569	1.0137	0.8641	0.074*	
C5	0.5181 (4)	0.9065 (2)	0.8138 (2)	0.0500 (7)	
H5	0.6435	0.8612	0.8065	0.060*	
C6	0.3275 (4)	0.87991 (19)	0.78990 (18)	0.0401 (6)	
C7	0.3258 (4)	0.77723 (18)	0.75853 (18)	0.0384 (6)	
C8	0.1961 (4)	0.77693 (19)	0.67287 (18)	0.0382 (6)	
C9	0.2085 (4)	0.67797 (19)	0.64472 (19)	0.0408 (6)	
C10	0.3516 (4)	0.5816 (2)	0.7011 (2)	0.0448 (7)	
H10	0.3601	0.5162	0.6817	0.054*	
C11	0.4824 (4)	0.57853 (19)	0.78513 (19)	0.0422 (6)	
C12	0.4641 (4)	0.6772 (2)	0.81216 (19)	0.0442 (6)	
H12	0.5491	0.6763	0.8693	0.053*	
C13	0.0786 (4)	0.67611 (19)	0.5518 (2)	0.0415 (6)	
C14	−0.1360 (4)	0.7119 (2)	0.5430 (2)	0.0552 (8)	
H14	−0.2056	0.7381	0.5966	0.066*	
C15	−0.2493 (5)	0.7095 (2)	0.4553 (3)	0.0704 (10)	
H15	−0.3942	0.7352	0.4498	0.085*	
C16	−0.1511 (6)	0.6698 (2)	0.3769 (3)	0.0705 (10)	
H16	−0.2285	0.6688	0.3179	0.085*	
C17	0.0605 (6)	0.6317 (2)	0.3851 (2)	0.0648 (9)	
H17	0.1276	0.6035	0.3321	0.078*	
C18	0.1756 (5)	0.6345 (2)	0.4715 (2)	0.0539 (7)	
H18	0.3203	0.6083	0.4764	0.065*	
C19	0.6392 (4)	0.4760 (2)	0.84286 (19)	0.0424 (6)	
C20	0.5946 (4)	0.3643 (2)	0.8704 (2)	0.0502 (7)	
H20	0.4625	0.3527	0.8526	0.060*	
C21	0.7426 (5)	0.2696 (2)	0.9238 (2)	0.0565 (8)	
H21	0.7098	0.1949	0.9416	0.068*	
C22	0.9375 (5)	0.2857 (2)	0.9504 (2)	0.0613 (8)	
H22	1.0369	0.2219	0.9869	0.074*	
C23	0.9862 (5)	0.3965 (3)	0.9231 (2)	0.0620 (8)	
H23	1.1186	0.4076	0.9410	0.074*	
C24	0.8376 (4)	0.4909 (2)	0.8691 (2)	0.0543 (7)	
H24	0.8716	0.5654	0.8502	0.065*	
C25	0.0839 (4)	0.94943 (18)	0.52404 (19)	0.0386 (6)	
C26	0.2771 (5)	0.9412 (2)	0.4569 (2)	0.0703 (9)	
H26A	0.3871	0.8873	0.5042	0.105*	
H26B	0.3228	1.0161	0.4244	0.105*	
H26C	0.2467	0.9148	0.3984	0.105*	
N1	0.0470 (3)	0.87680 (15)	0.61974 (15)	0.0412 (5)	

### Atomic displacement parameters (Å^2^)

**Table:**

	*U*^11^	*U*^22^	*U*^33^	*U*^12^	*U*^13^	*U*^23^
C1	0.0513 (18)	0.0484 (15)	0.0515 (16)	−0.0026 (14)	−0.0047 (13)	−0.0245 (13)
C2	0.062 (2)	0.0522 (17)	0.072 (2)	0.0061 (15)	−0.0097 (16)	−0.0359 (15)
C3	0.076 (2)	0.0555 (18)	0.071 (2)	−0.0078 (17)	−0.0073 (18)	−0.0380 (15)
C4	0.059 (2)	0.0664 (19)	0.073 (2)	−0.0183 (16)	−0.0041 (16)	−0.0367 (16)
C5	0.0514 (18)	0.0520 (16)	0.0505 (16)	−0.0042 (13)	−0.0021 (13)	−0.0238 (13)
C6	0.0505 (17)	0.0379 (13)	0.0292 (13)	−0.0020 (12)	−0.0051 (12)	−0.0096 (10)
C7	0.0475 (16)	0.0332 (13)	0.0324 (13)	−0.0002 (11)	−0.0063 (12)	−0.0104 (10)
C8	0.0453 (15)	0.0364 (13)	0.0309 (13)	−0.0007 (11)	−0.0049 (11)	−0.0112 (10)
C9	0.0497 (16)	0.0380 (13)	0.0349 (13)	−0.0042 (12)	−0.0040 (12)	−0.0136 (11)
C10	0.0569 (17)	0.0335 (13)	0.0420 (15)	0.0005 (12)	−0.0070 (13)	−0.0132 (11)
C11	0.0501 (16)	0.0370 (14)	0.0356 (14)	0.0035 (12)	−0.0063 (12)	−0.0114 (11)
C12	0.0525 (16)	0.0440 (14)	0.0346 (14)	0.0010 (12)	−0.0111 (12)	−0.0141 (11)
C13	0.0530 (17)	0.0331 (13)	0.0382 (14)	−0.0057 (12)	−0.0090 (13)	−0.0114 (11)
C14	0.0527 (18)	0.0529 (17)	0.0656 (19)	−0.0036 (14)	−0.0094 (15)	−0.0284 (14)
C15	0.061 (2)	0.0627 (19)	0.094 (3)	0.0026 (16)	−0.0332 (19)	−0.0357 (18)
C16	0.095 (3)	0.0565 (18)	0.064 (2)	−0.0099 (19)	−0.036 (2)	−0.0221 (16)
C17	0.091 (3)	0.066 (2)	0.0473 (18)	−0.0151 (18)	−0.0050 (17)	−0.0297 (15)
C18	0.0596 (19)	0.0569 (17)	0.0500 (17)	−0.0068 (14)	−0.0041 (15)	−0.0249 (13)
C19	0.0501 (17)	0.0432 (15)	0.0331 (13)	0.0049 (13)	−0.0060 (12)	−0.0164 (11)
C20	0.0566 (18)	0.0431 (15)	0.0452 (15)	0.0042 (13)	−0.0052 (13)	−0.0127 (12)
C21	0.071 (2)	0.0423 (15)	0.0488 (17)	0.0076 (14)	−0.0019 (15)	−0.0132 (13)
C22	0.066 (2)	0.0560 (18)	0.0494 (17)	0.0208 (16)	−0.0120 (15)	−0.0141 (14)
C23	0.0544 (19)	0.071 (2)	0.0600 (18)	0.0113 (16)	−0.0145 (15)	−0.0282 (15)
C24	0.0589 (19)	0.0503 (16)	0.0515 (17)	0.0052 (14)	−0.0088 (14)	−0.0194 (13)
C25	0.0428 (16)	0.0349 (13)	0.0367 (14)	0.0002 (11)	−0.0064 (12)	−0.0125 (11)
C26	0.060 (2)	0.0629 (18)	0.0619 (19)	0.0136 (16)	0.0013 (16)	−0.0005 (15)
N1	0.0475 (13)	0.0365 (11)	0.0378 (12)	0.0040 (10)	−0.0101 (10)	−0.0136 (9)

### Geometric parameters (Å, º)

**Table:**

C1—C2	1.374 (4)	C14—H14	0.9300
C1—C6	1.386 (3)	C15—C16	1.361 (4)
C1—H1	0.9300	C15—H15	0.9300
C2—C3	1.365 (4)	C16—C17	1.360 (4)
C2—H2	0.9300	C16—H16	0.9300
C3—C4	1.373 (4)	C17—C18	1.375 (4)
C3—H3	0.9300	C17—H17	0.9300
C4—C5	1.375 (4)	C18—H18	0.9300
C4—H4	0.9300	C19—C20	1.380 (4)
C5—C6	1.385 (4)	C19—C24	1.383 (4)
C5—H5	0.9300	C20—C21	1.381 (3)
C6—C7	1.479 (4)	C20—H20	0.9300
C7—C12	1.388 (3)	C21—C22	1.369 (4)
C7—C8	1.411 (3)	C21—H21	0.9300
C8—C9	1.399 (4)	C22—C23	1.379 (4)
C8—N1	1.422 (3)	C22—H22	0.9300
C9—C10	1.384 (3)	C23—C24	1.383 (3)
C9—C13	1.495 (4)	C23—H23	0.9300
C10—C11	1.384 (3)	C24—H24	0.9300
C10—H10	0.9300	C25—N1	1.268 (3)
C11—C12	1.384 (3)	C25—C26	1.479 (4)
C11—C19	1.482 (3)	C25—C25^i^	1.491 (4)
C12—H12	0.9300	C26—H26A	0.9600
C13—C14	1.373 (4)	C26—H26B	0.9600
C13—C18	1.391 (4)	C26—H26C	0.9600
C14—C15	1.379 (4)		
			
C2—C1—C6	121.2 (3)	C15—C14—H14	119.7
C2—C1—H1	119.4	C16—C15—C14	120.6 (3)
C6—C1—H1	119.4	C16—C15—H15	119.7
C3—C2—C1	120.6 (3)	C14—C15—H15	119.7
C3—C2—H2	119.7	C15—C16—C17	119.8 (3)
C1—C2—H2	119.7	C15—C16—H16	120.1
C2—C3—C4	119.5 (3)	C17—C16—H16	120.1
C2—C3—H3	120.3	C16—C17—C18	120.2 (3)
C4—C3—H3	120.3	C16—C17—H17	119.9
C3—C4—C5	119.8 (3)	C18—C17—H17	119.9
C3—C4—H4	120.1	C17—C18—C13	120.9 (3)
C5—C4—H4	120.1	C17—C18—H18	119.5
C4—C5—C6	121.8 (3)	C13—C18—H18	119.5
C4—C5—H5	119.1	C20—C19—C24	118.2 (2)
C6—C5—H5	119.1	C20—C19—C11	121.8 (3)
C1—C6—C5	117.0 (2)	C24—C19—C11	119.9 (2)
C1—C6—C7	123.3 (2)	C19—C20—C21	121.2 (3)
C5—C6—C7	119.7 (2)	C19—C20—H20	119.4
C12—C7—C8	117.7 (2)	C21—C20—H20	119.4
C12—C7—C6	119.0 (2)	C22—C21—C20	119.9 (3)
C8—C7—C6	123.2 (2)	C22—C21—H21	120.0
C9—C8—C7	120.2 (2)	C20—C21—H21	120.0
C9—C8—N1	121.0 (2)	C21—C22—C23	119.9 (3)
C7—C8—N1	118.7 (2)	C21—C22—H22	120.0
C10—C9—C8	118.9 (2)	C23—C22—H22	120.0
C10—C9—C13	119.4 (2)	C22—C23—C24	119.8 (3)
C8—C9—C13	121.5 (2)	C22—C23—H23	120.1
C9—C10—C11	122.7 (2)	C24—C23—H23	120.1
C9—C10—H10	118.7	C23—C24—C19	120.9 (3)
C11—C10—H10	118.7	C23—C24—H24	119.5
C12—C11—C10	117.0 (2)	C19—C24—H24	119.5
C12—C11—C19	120.8 (2)	N1—C25—C26	125.5 (2)
C10—C11—C19	122.2 (2)	N1—C25—C25^i^	116.6 (3)
C11—C12—C7	123.5 (2)	C26—C25—C25^i^	118.0 (3)
C11—C12—H12	118.2	C25—C26—H26A	109.5
C7—C12—H12	118.2	C25—C26—H26B	109.5
C14—C13—C18	117.8 (2)	H26A—C26—H26B	109.5
C14—C13—C9	122.6 (3)	C25—C26—H26C	109.5
C18—C13—C9	119.6 (3)	H26A—C26—H26C	109.5
C13—C14—C15	120.7 (3)	H26B—C26—H26C	109.5
C13—C14—H14	119.7	C25—N1—C8	121.1 (2)
			
C6—C1—C2—C3	0.1 (4)	C10—C9—C13—C14	133.0 (3)
C1—C2—C3—C4	0.3 (4)	C8—C9—C13—C14	−50.0 (3)
C2—C3—C4—C5	−0.1 (4)	C10—C9—C13—C18	−45.6 (3)
C3—C4—C5—C6	−0.4 (4)	C8—C9—C13—C18	131.3 (3)
C2—C1—C6—C5	−0.6 (4)	C18—C13—C14—C15	−1.8 (4)
C2—C1—C6—C7	177.1 (2)	C9—C13—C14—C15	179.6 (2)
C4—C5—C6—C1	0.7 (4)	C13—C14—C15—C16	1.0 (4)
C4—C5—C6—C7	−177.1 (2)	C14—C15—C16—C17	0.3 (5)
C1—C6—C7—C12	−139.5 (3)	C15—C16—C17—C18	−0.8 (4)
C5—C6—C7—C12	38.1 (3)	C16—C17—C18—C13	0.1 (4)
C1—C6—C7—C8	43.3 (4)	C14—C13—C18—C17	1.2 (4)
C5—C6—C7—C8	−139.1 (3)	C9—C13—C18—C17	180.0 (2)
C12—C7—C8—C9	0.3 (4)	C12—C11—C19—C20	142.3 (3)
C6—C7—C8—C9	177.6 (2)	C10—C11—C19—C20	−39.0 (4)
C12—C7—C8—N1	177.5 (2)	C12—C11—C19—C24	−38.7 (3)
C6—C7—C8—N1	−5.2 (4)	C10—C11—C19—C24	140.0 (3)
C7—C8—C9—C10	−0.9 (4)	C24—C19—C20—C21	0.7 (4)
N1—C8—C9—C10	−178.0 (2)	C11—C19—C20—C21	179.8 (2)
C7—C8—C9—C13	−177.9 (2)	C19—C20—C21—C22	0.1 (4)
N1—C8—C9—C13	5.0 (4)	C20—C21—C22—C23	−0.5 (4)
C8—C9—C10—C11	0.5 (4)	C21—C22—C23—C24	0.1 (4)
C13—C9—C10—C11	177.6 (2)	C22—C23—C24—C19	0.7 (4)
C9—C10—C11—C12	0.5 (4)	C20—C19—C24—C23	−1.1 (4)
C9—C10—C11—C19	−178.3 (2)	C11—C19—C24—C23	179.9 (2)
C10—C11—C12—C7	−1.1 (4)	C26—C25—N1—C8	0.2 (4)
C19—C11—C12—C7	177.7 (2)	C25^i^—C25—N1—C8	179.7 (2)
C8—C7—C12—C11	0.7 (4)	C9—C8—N1—C25	−81.2 (3)
C6—C7—C12—C11	−176.7 (2)	C7—C8—N1—C25	101.6 (3)

Symmetry code: (i) −*x*, −*y*+2, −*z*+1.

### Hydrogen-bond geometry (Å, º)

*Cg*1 is the centroid of the (C19–C24) phenyl ring.

**Table:**

*D*—H···*A*	*D*—H	H···*A*	*D*···*A*	*D*—H···*A*
C17—H17···*Cg*1^ii^	0.93	2.89	3.737 (5)	152

Symmetry code: (ii) −*x*+1, −*y*+1, −*z*+1.
